# Supplementary material for: Evolutionary origin of Hoxc13-dependent skin appendages in amphibians
Source: Nat Commun. 2024 Mar 18;15:2328. doi: 10.1038/s41467-024-46373-x (PMC10948813; doi:10.1038/s41467-024-46373-x)
Supplement: Supplementary file 1 — Supplementary Information [file 41467_2024_46373_MOESM1_ESM.pdf]

Supplementary information

Evolutionary origin of Hoxc13-dependent skin appendages in amphibians

Marjolein Carron, Attila Placido Sachslehner, Munevver Burcu Cicekdal, Inge Bruggeman, Suzan Demuynck, Bahar Golabi, Elfride De Baere, Wim Declercq, Erwin Tschachler, Kris Vleminckx, Leopold Eckhart

Supplementary methods

Preparation of riboprobes

Riboprobe templates that anneal at the end of the coding region and the 3'-untranslated regions of *X. tropicalis hoxc13, krt34,* and *krt59* were amplified by RT-PCR from toe cDNA using the following primer pairs: *hoxc13* (forward: 5'-CCACTTATGGAAAGCACCTTTCC-3' and reverse: 5'-GCAGGATTTGCCACTTGTTCC-3'), *krt34* (forward: 5'-CGTGGAAACACAACTAGGAAGC-3' and reverse: 5'-CCTTCACCACTTCTGACGACC-3'), and *krt59* (forward: 5'-GGAAGGTGCCATAACAGAGG-3' and reverse: 5'-GCTCAAGGCTAGATTGCTGG-3'). For axolotl, a riboprobe that anneals at the 3'-untranlsated region of *krt34* was amplified with the following primer pair: krt34 (forward: 5'-CCGAACAAATGCAGTCCACC-3' and reverse: 5'-GCTGAAGTGTTGTCTCATCCC-3'). The PCR products were ligated into a pGEM-T Easy vector (catalog number: A1380, Promega). Plasmids containing an insert were amplified in E. coli (catalog number: L2001, Promega). Plasmid DNA was isolated with the QIAprep spin miniprep kit 250 (catalog number: 27106, Qiagen) and sequenced (Microsynth) using SP6 primers to determine the orientation of the insert. Plasmids containing inserts in antisense and sense orientation were subjected to PCR with primers (forward: 5'-CGCCAGGGTTTTCCCAGTCACGAC-3' and reverse: 5'-CAGGAAACAGCTATGAC-3') to create an amplicon in which the insert is flanked by a T7 RNA-polymerase binding site. The PCR products were used as templates for in vitro transcription with the DIG RNA labeling mix (catalog number: 11175025910, Roche) and T7 RNA polymerase for transcription of antisense and sense probes, depending on the orientation of the insert. RNA probes were precipitated with 4 M LiCl (catalog number: L7026, Sigma-Aldrich) and ethanol overnight at -20°C, followed by subsequent washing steps with 70% and 100% ethanol and resuspension in RNase-free water. The riboprobes were stored at -80°C.

***In situ* hybridization**

In situ hybridization on formalin-fixed paraffin-embedded tissue sections was performed according to a published protocol [1]. Briefly, 5 µm thick paraffin sections were prepared with a Microm HM 335E microtome and mounted on super-adhesive slides (catalog number: 7608105, Menzel). The paraffin sections were melted at 58°C for one hour followed by deparaffinization in xylene (catalog number: 1330-20-7, Fisher Chemical) for 20 minutes. The

samples were further hydrated in a descending ethanol series. After washing in PBS, the samples were treated with 20 µg/ml proteinase K for 10 minutes at 37°C followed by another washing step in PBS. To neutralize charged probe binding, specimens were subsequently incubated for 10 min in 1% triethanolamine (catalog number: 90279, Sigma-Aldrich) in PBS, for 5 min in 1% triethanolamine with 0.15% acetic anhydride (catalog number: 21390293, Prolabo), and for 5 min in 1% triethanolamine with 0.3% acetic anhydride. After another wash step in PBS, samples were postfixed with 4% paraformaldehyde in PBS for 40 minutes followed by dehydration in ascending ethanol series. Samples were dried at room temperature for 1 hour, followed by incubation in hybridization buffer at 54°C for 2 hours. Sense and antisense riboprobes were pre-heated in hybridization buffer (4 M urea, catalog number: 0568, VWR; 5x saline sodium citrate (SSC), catalog number: 10541, Roth; 100 µg/ml heparin, catalog number: H3149, Sigma-Aldrich; 5 mM EDTA, catalog number: 80401, Roth; 1x Denhardt's block reagent, catalog number: D2531, Sigma-Aldrich; 100 µg/ml salmon sperm DNA, catalog number: 201190, Agilent; 5% dextran sulfate, catalog number: D8906, Sigma-Aldrich) at 85°C for 10 minutes before they were added to the samples at a concentration of 2 ng/µl. Hybridization took place overnight at 54°C. To remove unbound probes, samples were washed once with a solution containing 4 M urea and 4x SSC, once with a solution containing 4 M urea and 2x SSC, and once with a solution containing 4 M urea and 1x SSC at 58°C for 15 minutes each, followed by one wash with 1x SSC at 37°C for 15 minutes. To prevent non-specific anti-digoxigenin antibody binding, specimens were incubated for two hours in 0.1 M maleic acid buffer (MAB, catalog number: M0375, Sigma-Aldrich), pH 7.5 containing 2% bovine serum albumin (BSA, catalog number: A3608-500, Sigma-Aldrich). Afterwards, specimens were incubated with an anti-digoxigenin antibody conjugated to the alkaline phosphatase enzyme (1:5000, catalog number: 11093274910, Roche) in 2% MAB block solution overnight at 4°C. A buffer, suitable for alkaline phosphatase enzyme activity (0.5 M Tris pH 9.5; 0.5 M NaCl) was prepared and samples were washed twice in alkaline phosphatase buffer for 15 minutes at room temperature. The staining was developed with alkaline phosphatase buffer supplemented with 0.05 M $MgCl_2$, 3.75 µl/ml 5-bromo-4-chloro-3- indolyl phosphate (BCIP) (catalog number: 11383221001, Roche) and 5 µl/ml nitroblue tetrazolium (NBT) (catalog number: 11383213001, Roche). Sections were mounted with Aquatex (catalog number: 108562, Sigma Aldrich) and photographs were taken with an Olympus UC-90 camera on an Olympus BX63 microscope using the software cellSense Dimension (version: 2.3.18987.0, https://www.olympus-lifescience.com/en/software/cellsens/).

Protein quantification by mass spectrometry-based proteomics

Samples for proteomic analysis were dissected freshly and placed immediately in 200 µl lysis buffer, which consisted of 30 mM Tris, 7 M urea (catalog number: 0568, VWR), 2 M thiourea (catalog number: T7875, Sigma-Aldrich), 4% CHAPSO (catalog number: 28304, Pierce). 0.2 M dithiothreitol (DTT) was added to the samples. After incubation at 70°C for 3 hours the samples were homogenized with a homogenizer (Precellys, VWR), centrifuged at 18000 g for 15 minutes at 4°C, and the supernatant was collected. The pellet was sonicated with a sonicator (Hielscher Ultrasound Technology) twice for 30 seconds at an amplitude of 100, and centrifuged again at 18000 g for 15 minutes at 4°C. The resulting supernatant was pooled with the one from the initial homogenization and stored at -80°C until analysis was started. For each sample, the protein lysate was topped up to 500 µl with 8 M urea (catalog number: 2317.1, Roth) in 50 mM Tris and loaded onto a Pall 10 kDa filter performing a FASP protocol [2]. The solution was centrifuged twice for 20 min at 10000 g. The cysteine residues of the proteins were reduced with 200 mM DTT (37°C, 30 min, catalog number: 6908.3, Roth) and alkylated with 500 mM iodoacetamide (IAA, catalog number: I1149, Sigma) at 37°C for 30 min on the filter. Before liquid chromatography–mass spectrometry (LC-MS) analysis, peptide extracts were desalted and cleaned up using C18 spin columns (catalog number: 89870, Thermo-Fisher) according to the manufacturer's protocol. The dried peptides were dissolved in 15 µl 0.1% TFA (catalog number: 10723857, Fisher Scientific). Peptides were separated on a nano-HPLC Ultimate 3000 RSLC system (Dionex). Sample pre-concentration and desalting was accomplished with a 5 mm Acclaim PepMap µ-Precolumn (300 µm inner diameter, 5 µm particle size, and 100 Å pore size) (Dionex). For sample loading and desalting, 2% acetonitrile (ACN) in ultra-pure LC-MS grade H_2O with 0.05% TFA was used as a mobile phase with a flow rate of 5 µl/min. Separation of peptides was performed on a 25 cm Acclaim PepMap C18 column (75 µm inner diameter, 2 µm particle size, and 100 Å pore size, OD010C34, Pall) with a flow rate of 300 nl/min. The gradient started with 4% mobile phase B (80% ACN with 0.08% formic acid, catalog number: 28905, Pierce) for 7 min, increased to 31% in 60 min and to 44% in additional 5 min. This was followed by a washing step with 95% phase B. Mobile phase A consisted of ultra pure LC-MS grade H_2O with 0.1% formic acid. For mass spectrometric analysis, the LC was directly coupled to a high-resolution Q Exactive HF Orbitrap mass spectrometer.

MS full scans were performed in the ultrahigh-field Orbitrap mass analyzer in ranges m/z 350−2000 with a resolution of 60 000, the maximum injection time (MIT) was 50 ms and the automatic gain control (AGC) was set to 3e^6. The top 10 intense ions were subjected to Orbitrap for further fragmentation via high energy collision dissociation (HCD) activation over a mass range between m/z 200 and 2000 at a resolution of 15 000 with the intensity threshold at 4e^3. Ions with charge states +1, +7,+8 and >+8 were excluded. Normalized collision energy (NCE) was set at 28. For each scan, the AGC was set at 5e^4 and the MIT was 50 ms. Dynamic exclusion of precursor ion masses over a time window of 30 s was used to suppress repeated peak fragmentation.

Database search was performed using the Proteome Discoverer Software 2.4.1.15 (Thermo Fisher Scientific) with the following settings: -Protein Database: UP_Xenopus_tropicalis_tx8364_230220.fasta (www.unipot.org, taxonomy ID 8364), crap.fasta (https://www.thegpm.org/crap/), - Enzyme name: Trypsin (full), - Max. missed cleavage sites: 2, - Precursor mass tolerance: 10 ppm, - Fragment mass tolerance: 0.02 Da, - Dynamic modification: Oxidation / +15.995 Da (M), - Dynamic modification: Deamidation / +0.984 Da (N,Q), - N-terminal modification: Gln->pyro-Glu / -17.027 Da (Q), - N-terminal modification: Acetyl / +42.011 Da (N-Terminus), - N-terminal modification: Met-loss / -131.040 Da (M), - N-terminal modification: Met-loss+Acetyl / -89.030 Da (M), - Static modification: Carbamidomethyl / +57.021 Da (C), Decoy Database search: - Target FDR (strict): 0.01, - Target FDR (relaxed): 0.05, - Validation based on: q-Value. Evaluation of protein abundances including normalisation to total peptide amount was performed in Proteome Discoverer software (version 2.4.1.15, Thermo Fisher Scientific).

RNA-seq and data analysis

Sequencing libraries were prepared using the NEB Next Poly(A) mRNA Magnetic Isolation Module and the NEBNext Ultra™ II Directional RNA Library Prep Kit for Illumina according to the manufacturer's protocols (New England Biolabs). Libraries were QC-checked on a Bioanalyzer 2100 (Agilent) using a High Sensitivity DNA Kit for correct insert size and quantified using Qubit dsDNA HS Assay (Invitrogen). Pooled libraries were sequenced on a NextSeq500 instrument (Illumina) in 1x75bp single-end sequencing mode. 33-37 million reads per sample were generated. Salmon (version: 1.10.1) [3] was used to map the reads to the *X. tropicalis* reference genome UCB_Xtro_10.0, NCBI (accession number: GCA_000004195.4)

and to calculate the read counts. Differential gene expression analysis was performed with the Python implemented module PyDESeq2 [4] in Python (version: 3.10). To assess statistical significance, the Wald test, followed by the Benjamini Hochberg correction for multiple testing, was performed [5].

Supplementary references

1. Mlitz, V., Hermann, M., Buchberger, M., Tschachler, E. & Eckhart L. The trichohyalin-like protein scaffoldin is expressed in the multilayered periderm during development of avian beak and egg tooth. *Genes (Basel)* **12**, 248 (2021).

2. Wisniewski, J. R., Zougman, A., Nagaraj, N. & Mann, M. Universal sample preparation method for proteome analysis. *Nat. Methods* **6**, 359-362 (2009).

3. Patro, R., Duggal, G., Love, M.I., Irizarry, R.A. & Kingsford, C. Salmon provides fast and bias-aware quantification of transcript expression. *Nat. Methods* **14**, 417-419 (2017).

4. Muzellec, B., Teleńczuk, M., Cabeli, V. & Andreux, M. PyDESeq2: a python package for bulk RNA-seq differential expression analysis. *Bioinformatics*. **39**, btad547 (2023).

5. Chen, Z. et al. Statistical methods on detecting differentially expressed genes for RNA-seq data. *BMC Syst. Biol.* **5**, Suppl 3(Suppl 3):S1 (2011).

Supplementary figures

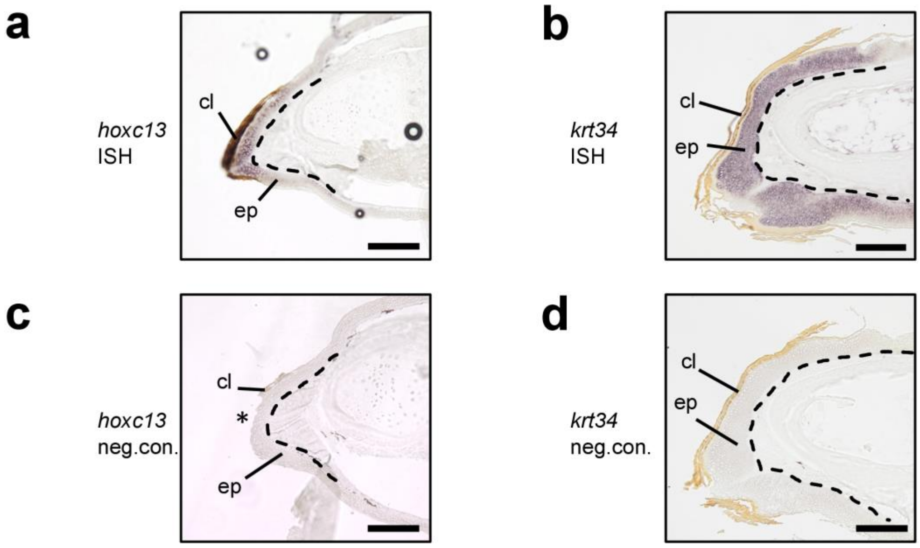

Supplementary Figure 1. *In situ* hybridization of *hoxc13* and *krt34* mRNA in clawed toes of *Xenopus tropicalis*. a, c mRNA *in situ* hybridization (ISH) using antisense probes for *hoxc13* (**a**) and *krt34* (**b**) on sections of hind limb inner (HI) toes of *X. tropicalis*. Hybridizations with sense probes for *hoxc13* (**c**) and *krt34* (**d**) served as negative controls (neg. con.). The plane of the sections was oblique in **b** and **d**. Dashed lines mark the junction of epidermis and dermis. The cornified claws were less well-preserved on these sections than on sections of the experiment shown in Fig. 3. An asterisk marks the detachment of the cornified claw during tissue sectioning. Age of the frogs: 4 months (**a**, **c**), 7 months (**b**, **d**). cl = claw, ep = epidermis. Scale bars: 200 µm.

a

hoxc13 mutations: nucleotide sequences

```
guide RNA                CCAGGCAATGCACGGCTCCC
wildtype       TCTCCAGAAGCCGCCAGGCAATGCACGGCTCCCCCGTCTTCTTCC
                S  P  E  A  A  R  Q  C  T  A  P  P  S  S  S

Deletions
mutation 1     TCTCCAGAAGCC-----GCAATGCACGGCTCCCCCGTCTTCTTCC
                S  P  E  A        A  M  H  G  S  P  V  F  F

mutation 2     TCTCCAGAAGCCGCC--GCAATGCACGGCTCCCCCGTCTTCTTCC
                S  P  E  A  A     A  M  H  G  S  P  V  F  F

mutation 3     TCTCCAGAAGCCGCC-----ATGCACGGCTCCCCCGTCTTCTTCC
                S  P  E  A  A        M  H  G  S  P  V  F  F

mutation 4     TCTCCAG-----------------ACGGCTCCCCCGTCTTCTTCC
                S  P                    D  G  S  P  V  F  F

mutation 5     TCTCCAGAAGCCGC----CAATGCACGGCTCCCCCGTCTTCTTCC
                S  P  E  A  A        N  A  R  L  P  R  L  L  P

Insertion
mutation 6     TCTCCAGAAGCCGCCAGAAGCAATGCACGGCTCCCCCGTCTTCTT
                S  P  E  A  A  R  S  N  A  R  L  P  R  L  L
```

b

Impact of mutations on the Hoxc13 protein structure

```
                                      <                                   HoxA13_N domain
wt     MTTSLILHPHWADTLMYVYETSPNEKNPRKSPAMEGLSGNCASSHCRDFISHPALGRHSSAIGSHQGPVYTDITSPEAAR-QCTAPPSSSNASLGYAYPFGSS
Mut 1  MTTSLILHPHWADTLMYVYETSPNEKNPRKSPAMEGLSGNCASSHCRDFISHPALGRHSSAIGSHQGPVYTDITSPEAA-MHGSPVFFQRLPGLCLPIWKQLL
Mut 2  MTTSLILHPHWADTLMYVYETSPNEKNPRKSPAMEGLSGNCASSHCRDFISHPALGRHSSAIGSHQGPVYTDITSPEAAAMHGSPVFFQRLPGLCLPIWKQLL
Mut 3  MTTSLILHPHWADTLMYVYETSPNEKNPRKSPAMEGLSGNCASSHCRDFISHPALGRHSSAIGSHQGPVYTDITSPEAA-MHGSPVFFQRLPGLCLPIWKQLL
Mut 4  MTTSLILHPHWADTLMYVYETSPNEKNPRKSPAMEGLSGNCASSHCRDFISHPALGRHSSAIGSHQGPVYTDITSPD-----GSPVFFQRLPGLCLPIWKQLL
Mut 5  MTTSLILHPHWADTLMYVYETSPNEKNPRKSPAMEGLSGNCASSHCRDFISHPALGRHSSAIGSHQGPVYTDITSPEAA--NARLPRLLPTPPWAMPTHLEAA
Mut 6  MTTSLILHPHWADTLMYVYETSPNEKNPRKSPAMEGLSGNCASSHCRDFISHPALGRHSSAIGSHQGPVYTDITSPEAARSNARLPRLLPTPPWAMPTHLEAA

                      HoxA13_N domain            >
wt     YYGCRLSQSHNVNLHQKPCSYHPAEKYPEPSNPLPSEEFSSRAKEFAFYPSFASSYQAVPGYLDMSVVPGISSHPEPRHDTLLSMEGYQHWALPNGWDGQVYC
Mut 1  RLQALSVPQRQLTPETLLLPSGREIPRAQQPLAQRRILLQGQGICFLP*------------------------------------------------------
Mut 2  RLQALSVPQRQLTPETLLLPSGREIPRAQQPLAQRRILLQGQGICFLP*------------------------------------------------------
Mut 3  RLQALSVPQRQLTPETLLLPSGREIPRAQQPLAQRRILLQGQGICFLP*------------------------------------------------------
Mut 4  RLQALSVPQRQLTPETLLLPSGREIPRAQQPLAQRRILLQGQGICFLP*------------------------------------------------------
Mut 5  ITAAGSLSPTTSTYTRNPAPTIRPRNTPSPATPCPAKNSPPGPRNLLFTLVLPAPTRRSLVTWTCQ*------------------------------------
Mut 6  ITAAGSLSPTTSTYTRNPAPTIRPRNTPSPATPCPAKNSPPGPRNLLFTLVLPAPTRRSLVTWTCQ*------------------------------------

                                      <                         Homeobox domain                  >
wt     SKDQSQSGHLWKAPFPDVVPLQPEVSNYRRGRKKRVPYTKIQLKELEKEYAASKFITKEKRRRISTATSLSERQVTIWFQNRRVKEKKVVTKCKTAHLHNT
Mut 1  -----------------------------------------------------------------------------------------------------
Mut 2  -----------------------------------------------------------------------------------------------------
Mut 3  -----------------------------------------------------------------------------------------------------
Mut 4  -----------------------------------------------------------------------------------------------------
Mut 5  -----------------------------------------------------------------------------------------------------
Mut 6  -----------------------------------------------------------------------------------------------------
```

Supplementary Figure 2. *hoxc13* mutations and the potential impact on the Hoxc13 protein. a Nucleotide sequences of wildtype and mutant *Xenopus tropicalis hoxc13* in the region targeted by the guide RNA. The wildtype sequence corresponds to the complementary sequence of nucleotides 146200798-146200842, GenBank accession number NC_030678.2). Deletions (represented by dashes) and insertions are shown with red fonts. The amino acid sequence encoded by each nucleotide sequence are also shown. **b** Impact of the mutations on the structure of Hoxc13. The amino acid sequences of wildtype (wt) and mutant (Mut) Hoxc13 proteins are aligned. Amino acid sequences encoded downstream of out-of-frame mutations are shown in red. Dashes were introduced in the wt and mutant sequences to improve the alignment. Asterisks denote premature ends of the protein. The positions of protein domains are indicated above the alignment.

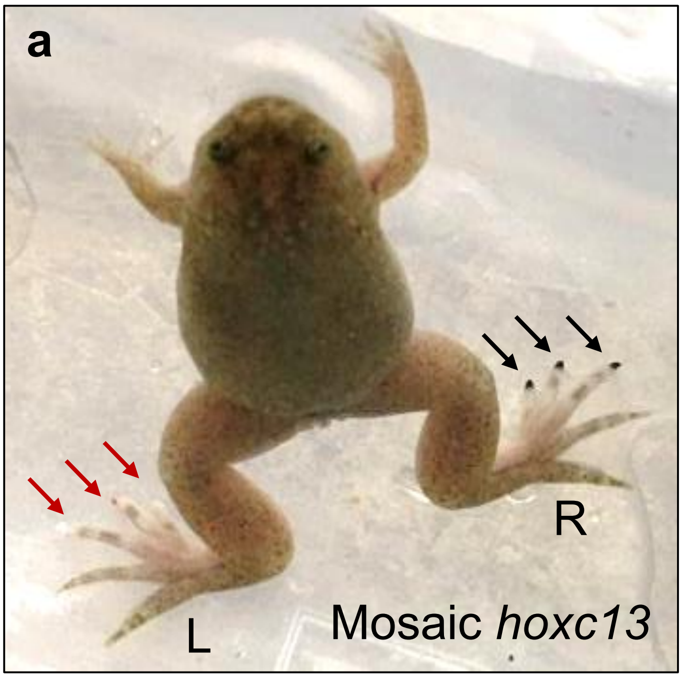

b

Frog	Leg	Phenotype	*hoxc13*	Sequence	Hoxc13 protein
Wildtype	L, R	claws	alleles 1, 2	TCTCCAGAAGCCGCCAGGCAATGCACGGCTCCCCCGTCTTCTTCC<br>S P E A A R Q C T A P P S S S	full-length wildtype
Mosaic	L	no claws	allele 1	TCTCCAGAAGCC------GCAATGCACGGCTCCCCCGTCTTCTTCC<br>S P E A A M H G S P V F F	truncated
			allele 2	TCTCCAGAAGCCGC----CAATGCACGGCTCCCCCGTCTTCTTCC<br>S P E A A N A R L P R L L P	truncated
Mosaic	R	claws	allele 1	TCTCCAGAAGCCGCCATGCAATGCACGGCTCCCCCGTCTTCTTCC<br>S P E A A M Q C T A P P S S S	full-length R>M
			allele 2	TCTCCAGAAGCCGC----CAATGCACGGCTCCCCCGTCTTCTTCC<br>S P E A A N A R L P R L L P	truncated

Supplementary Figure 3. Mutations of *hoxc13* in a mosaic mutant frog lacking claws on the toes of one hindlimb. a Phenotype of the mosaic mutant frog frog in the F0 generation. Black arrows indicate hindlimb inner (HI) toes with claws. Red arrows indicate hindlimb inner (HI) toes without claws. **b** Sequence of hoxc13 alleles in the gene region also shown in Supplementary figure 2. The sequences were determined by Sanger sequencing of PCR products. Note that HI toes lacking claws (left hindlimb) have out-of-frame mutations in both alleles, and HI toes bearing claws (right hindlimb) have one allele with an out-of-frame mutation and one allele with a point mutation that leads to amino acid substitution R80M, but allows the synthesis of full length Hoxc13 protein. L, left; R, right.

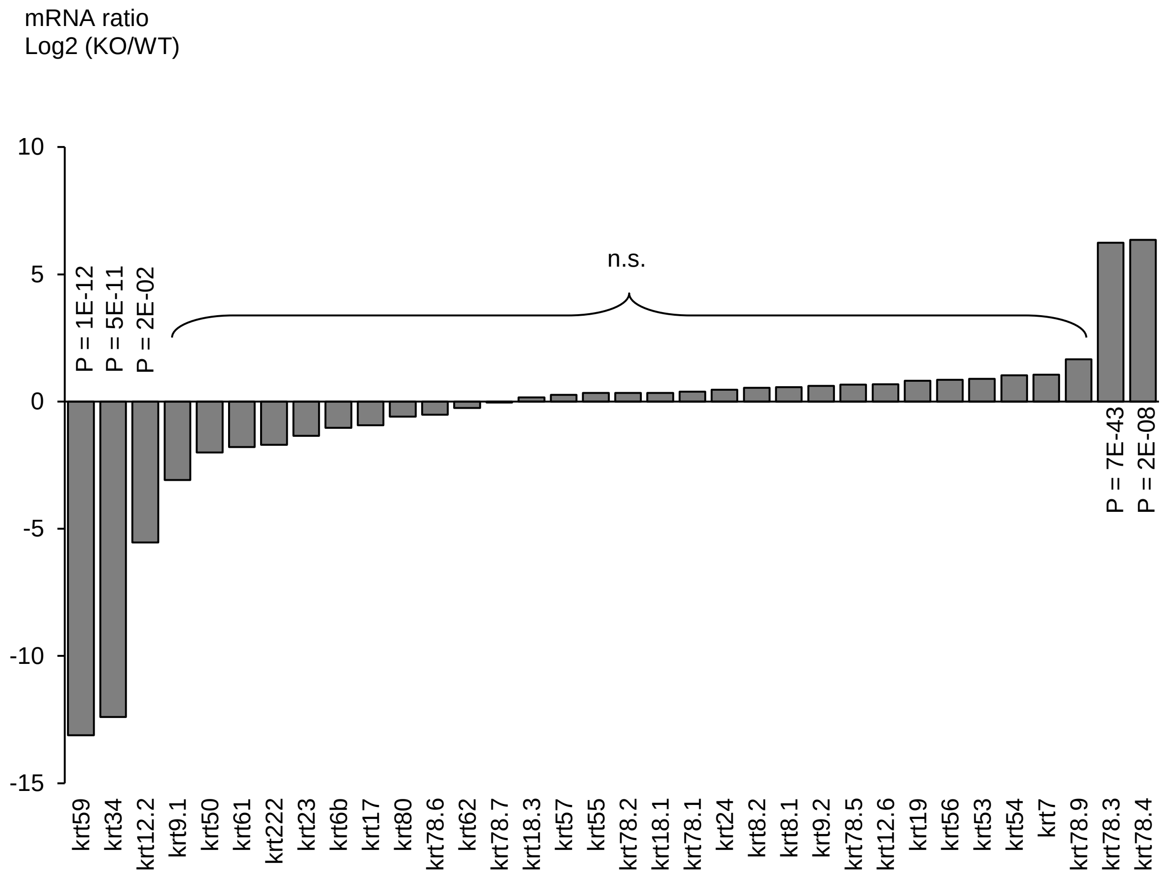

Supplementary Figure 4. Keratin gene expression in *hoxc13* wildtype (WT) and knockout (KO) hindlimb inner (HI) toes. Keratin mRNA levels were determined by RNA-seq analysis of RNA extracted from HI toes (n=3 biological replicates per group). The samples were obtained from two wildtype frogs, two *hoxc13* KO frogs and separately from the genetically distinct hindlimbs of a mosaic mutant frog (Supplementary Figure 3). The Wald test, followed by the Benjamini Hochberg correction for multiple testing, was used for statistical analysis. Bars show the Log2 ratios of expression levels in KO versus WT samples. Adjusted P values are indicated when the expression levels in KO and WT were significantly different. n.s., not significant.

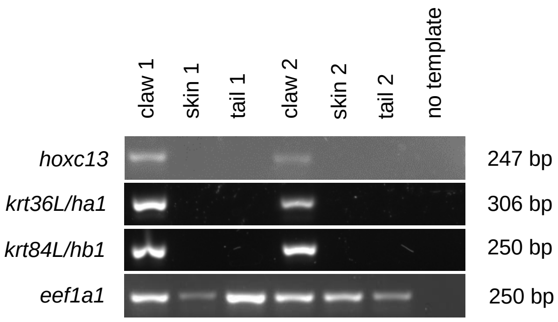

Supplementary Figure 5. Hair keratin homologs are co-expressed with *hoxc13* in the claws of the green anole lizard (*Anolis carolinensis*). The expression of *hoxc13* and hair keratin homologs *krt36L/ha1* (type I keratin) and *krt84L/hb1* (type II keratin) (Eckhart et al. 2008) was determined by RT-PCR. Claw samples contain the tissue underneath the cornified claws. Skin from the belly and tail of adult lizards was investigated for comparison. The housekeeping gene *eef1a1* was used as control. Tissues from two anole lizards were investigated. Lengths of PCR products are shown on the right. bp, base pairs.

a

>krt34_prom_wt_Gblock
CTGAGCTCGCTAGCCTCGAGGGGAGGGTGCATCGGTT**TTTATAAA**AGAAATAAAATTGTATACTCAGA
AAACCAACCACTAACCCAAAATGGTAAATTTTATAGCTAGTGCTTTATGGCGT**TTAATAAA**AAAACGT
CATATTATGTGTTTTTGTATCAAGACTGTAGACTGTCAACACCCACGGCTTTGATATAAAAGGCACA
ACGCAAGCCGATGAGTTGCAAGAGACATTTGTTGGACACATCGAACTAGAGGTTTCCAGTCACCACCG
GATATCAAGATCTGGCCTCGGCG

b

>krt34_prom_mut_Gblock
CTGAGCTCGCTAGCCTCGAGGGGAGGGTGCATCGGTT**T***GCCG***AAA**AGAAATAAAATTGTATACTCAGA
AAACCAACCACTAACCCAAAATGGTAAATTTTATAGCTAGTGCTTTATGGCGT**T***GCCG***AAA**AAAACGT
CATATTATGTGTTTTTGTATCAAGACTGTAGACTGTCAACACCCACGGCTTTGATATAAAAGGCACA
ACGCAAGCCGATGAGTTGCAAGAGACATTTGTTGGACACATCGAACTAGAGGTTTCCAGTCACCACCG
GATATCAAGATCTGGCCTCGGCG

c

>krt59_prom_wt_Gblock
CTGAGCTCGCTAGCCTCGAGGGCATTTAAAATGCTGT**TTTATAGA**AGGGAAAATAACCAGCAATAATG
CTTGTCATCTTTTGTAATTTGGTAGGTGTGTTGCTTTTTTGGTATCTGTAAATGGAGTAACAATGACA
TGCATTGCCAGCACTTTAGGCTAGTAAAATACAATGACAGGGTCTTTTCTGTTCTACGAGAGGACCAA
ACCCATTTGTCTGAGTATAAAAAGGAGAAGGTGGCTGCTCTTTACTCACAAGGACTTGCTTGAGCTTC
AGGTGTTGTCTGCTTTATTTCTCTATCTCTTTACCCAAGCATCAGGATATCAAGATCTGGCCTCGGCG

d

>krt59_prom_mut_Gblock
CTGAGCTCGCTAGCCTCGAGGGCATTTAAAATGCTGT**T***GCCG***AGA**AGGGAAAATAACCAGCAATAATG
CTTGTCATCTTTTGTAATTTGGTAGGTGTGTTGCTTTTTTGGTATCTGTAAATGGAGTAACAATGACA
TGCATTGCCAGCACTTTAGGCTAGTAAAATACAATGACAGGGTCTTTTCTGTTCTACGAGAGGACCAA
ACCCATTTGTCTGAGTATAAAAAGGAGAAGGTGGCTGCTCTTTACTCACAAGGACTTGCTTGAGCTTC
AGGTGTTGTCTGCTTTATTTCTCTATCTCTTTACCCAAGCATCAGGATATCAAGATCTGGCCTCGGCG

Supplementary Figure 6. Sequences of wildtype and mutated keratin promoters for promoter activation assays. Sequences of the proximal promoters (marked by blue fonts) of *krt34*, corresponding to GenBank accession number NC_030686.2, nucleotides 7761154-7761404 (**a, b**), and *krt59*, corresponding to GenBank accession number NC_030678.2, nucleotides 148109284-148109578 (**c, d**), of *X. tropicalis* were synthesized as parts of Gblocks (Integrated DNA Technologies, IDT). The promoter sequences are flanked by sequences required for cloning into the pGL4.10[luc2] plasmid (Promega). wt = wild type, mut = mutated. Predicted Hoxc13 binding sites are marked by bold fonts with core motifs being underlined (**a**, **c**). Mutations of core motifs are marked by italics (**b**, **d**). TATA boxes are marked by double underlines.

Supplementary Table 1. Type I keratins used for phylogenetic analysis

Species (simplified name)	Species (scientific name)	Gene*	Accession number**	Genome version
human	*Homo sapiens*	KRT24	NP_061889	GRCh38.p14
human	*Homo sapiens*	KRT25	XP_011522716	GRCh38.p14
human	*Homo sapiens*	KRT26	NP_853517	GRCh38.p14
human	*Homo sapiens*	KRT27	NP_853515	GRCh38.p14
human	*Homo sapiens*	KRT28	NP_853513	GRCh38.p14
human	*Homo sapiens*	KRT10	NP_001366295	GRCh38.p14
human	*Homo sapiens*	KRT12	NP_000214	GRCh38.p14
human	*Homo sapiens*	KRT20	NP_061883	GRCh38.p14
human	*Homo sapiens*	KRT23	XP_047291682	GRCh38.p14
human	*Homo sapiens*	KRT39	NP_998821	GRCh38.p14
human	*Homo sapiens*	KRT40	NP_872303	GRCh38.p14
human	*Homo sapiens*	KRT33a	NP_004129	GRCh38.p14
human	*Homo sapiens*	KRT33b	NP_002270	GRCh38.p14
human	*Homo sapiens*	KRT34	NP_001372943	GRCh38.p14
human	*Homo sapiens*	KRT31	NP_002268	GRCh38.p14
human	*Homo sapiens*	KRT37	NP_003761	GRCh38.p14
human	*Homo sapiens*	KRT38	NP_006762	GRCh38.p14
human	*Homo sapiens*	KRT32	NP_002269	GRCh38.p14
human	*Homo sapiens*	KRT35	NP_002271	GRCh38.p14
human	*Homo sapiens*	KRT36	NP_003762	GRCh38.p14
human	*Homo sapiens*	KRT13	NP_705694	GRCh38.p14
human	*Homo sapiens*	KRT15	NP_002266	GRCh38.p14
human	*Homo sapiens*	KRT19	NP_002267	GRCh38.p14
human	*Homo sapiens*	KRT9	NP_000217	GRCh38.p14
human	*Homo sapiens*	KRT14	NP_000517	GRCh38.p14
human	*Homo sapiens*	KRT16	NP_005548	GRCh38.p14
human	*Homo sapiens*	KRT17	NP_000413	GRCh38.p14
frog	*Xenopus tropicalis*	krt24	XP_002940698	UCB_Xtro_10.0
frog	*Xenopus tropicalis*	krt50	XP_031750197	UCB_Xtro_10.0
frog	*Xenopus tropicalis*	krt51	XP_031750195	UCB_Xtro_10.0
frog	*Xenopus tropicalis*	krt53	NP_001121485	UCB_Xtro_10.0
frog	*Xenopus tropicalis*	krt54	XP_002940703	UCB_Xtro_10.0
frog	*Xenopus tropicalis*	krt55	NP_001011224	UCB_Xtro_10.0
frog	*Xenopus tropicalis*	krt56	XP_004918844	UCB_Xtro_10.0
frog	*Xenopus tropicalis*	krt57	XP_031750559	UCB_Xtro_10.0
frog	*Xenopus tropicalis*	krt12.6	XP_002940696	UCB_Xtro_10.0
frog	*Xenopus tropicalis*	krt12.5	NP_001116496	UCB_Xtro_10.0
frog	*Xenopus tropicalis*	krt12.4	NP_001120056	UCB_Xtro_10.0
frog	*Xenopus tropicalis*	krt12.3	NP_001016659	UCB_Xtro_10.0
frog	*Xenopus tropicalis*	krt20	XP_031750289	UCB_Xtro_10.0
frog	*Xenopus tropicalis*	krt12.2	XP_004918756	UCB_Xtro_10.0
frog	*Xenopus tropicalis*	krt12.1	XP_031750293	UCB_Xtro_10.0
frog	*Xenopus tropicalis*	krt12.7	XP_031750291	UCB_Xtro_10.0

frog	*Xenopus tropicalis*	krt23	XP_031750290	UCB_Xtro_10.0
frog	*Xenopus tropicalis*	krt34	XP_002939082	UCB_Xtro_10.0
frog	*Xenopus tropicalis*	krt15.2	XP_002939083	UCB_Xtro_10.0
frog	*Xenopus tropicalis*	krt15.1	XP_004918754	UCB_Xtro_10.0
frog	*Xenopus tropicalis*	krt19	XP_012827034	UCB_Xtro_10.0
frog	*Xenopus tropicalis*	krt9.2	XP_012808156	UCB_Xtro_10.0
frog	*Xenopus tropicalis*	krt9.1	XP_002939076	UCB_Xtro_10.0
frog	*Xenopus tropicalis*	krt17	XP_002939077	UCB_Xtro_10.0
frog	*Xenopus tropicalis*	krt70	NP_001006687	UCB_Xtro_10.0
axolotl	*Ambystoma mexicanum*	krt24.1	AMEX60DD301010132.1	AmexG_v6.0-DD
axolotl	*Ambystoma mexicanum*	krt-T1-1	AMEX60DD301010128.1	AmexG_v6.0-DD
axolotl	*Ambystoma mexicanum*	krt12.6L	AMEX60DD201010125.1	AmexG_v6.0-DD
axolotl	*Ambystoma mexicanum*	krt12.5L	AMEX60DD201010124.2	AmexG_v6.0-DD
axolotl	*Ambystoma mexicanum*	krt-T1-2	AMEX60DD201010122.2	AmexG_v6.0-DD
axolotl	*Ambystoma mexicanum*	krt-T1-3	AMEX60DD201010120.31	AmexG_v6.0-DD
axolotl	*Ambystoma mexicanum*	krt23.1	AMEX60DD301010109.1	AmexG_v6.0-DD
axolotl	*Ambystoma mexicanum*	krt34	AMEX60DD201010106.2	AmexG_v6.0-DD
axolotl	*Ambystoma mexicanum*	krt15.2	AMEX60DD201010104.2	AmexG_v6.0-DD
axolotl	*Ambystoma mexicanum*	krt15.1	AMEX60DD101010103.1	AmexG_v6.0-DD
axolotl	*Ambystoma mexicanum*	krt19	AMEX60DD201010100.1	AmexG_v6.0-DD
axolotl	*Ambystoma mexicanum*	krt9.1	AMEX60DD301010096.2	AmexG_v6.0-DD
axolotl	*Ambystoma mexicanum*	krt17	AMEX60DD301010094.1	AmexG_v6.0-DD
caecilian	*Rhinatrema bivittatum*	krt24L1	XP_029429144.1	aRhiBiv1.1
caecilian	*Rhinatrema bivittatum*	krt24L2	XP_029428948.1	aRhiBiv1.1
caecilian	*Rhinatrema bivittatum*	krt24L3	XP_029428945.1	aRhiBiv1.1
caecilian	*Rhinatrema bivittatum*	krt24L4	XP_029428946.1	aRhiBiv1.1
caecilian	*Rhinatrema bivittatum*	krt-T1-1	XP_029428109.1	aRhiBiv1.1
caecilian	*Rhinatrema bivittatum*	krt-T1-2	XP_029428944.1	aRhiBiv1.1
caecilian	*Rhinatrema bivittatum*	krt-T1-3	XP_029428943.1	aRhiBiv1.1
caecilian	*Rhinatrema bivittatum*	krt-T1-4	XP_029428942.1	aRhiBiv1.1
caecilian	*Rhinatrema bivittatum*	krt20	XP_029428107.1	aRhiBiv1.1
caecilian	*Rhinatrema bivittatum*	krt12.1	XP_029429601.1	aRhiBiv1.1
caecilian	*Rhinatrema bivittatum*	krt12.7	XP_029430032.1	aRhiBiv1.1
caecilian	*Rhinatrema bivittatum*	krt23	XP_029428388.1	aRhiBiv1.1
caecilian	*Rhinatrema bivittatum*	krt15	XP_029428387.1	aRhiBiv1.1
caecilian	*Rhinatrema bivittatum*	krt19L	XP_029428371.1	aRhiBiv1.1
caecilian	*Rhinatrema bivittatum*	krt17L	XP_029429097.1	aRhiBiv1.1
caecilian	*Rhinatrema bivittatum*	krt70	XP_029429322.1	aRhiBiv1.1
lungfish	*Protopterus annectens*	KRT-T1-2	XP_043910934.1	GCF_019279795.1
lungfish	*Protopterus annectens*	KRT-T1-3	XP_043910932.1	GCF_019279795.1
lungfish	*Protopterus annectens*	KRT-T1-4	XP_043910931.1	GCF_019279795.1
lungfish	*Protopterus annectens*	KRT-T1-5	XP_043910930.1	GCF_019279795.1
lungfish	*Protopterus annectens*	KRT-T1-6	XP_043910928.1	GCF_019279795.1
lungfish	*Protopterus annectens*	KRT12L1	XP_043910921.1	GCF_019279795.1
lungfish	*Protopterus annectens*	KRT12L2	XP_043910914.1	GCF_019279795.1
lungfish	*Protopterus annectens*	KRT-T1-7	XP_043910913.1	GCF_019279795.1
lungfish	*Protopterus annectens*	KRT-T1-8	XP_043910911.1	GCF_019279795.1

lungfish	*Protopterus annectens*	KRT-T1-9	XP_043910907.1	GCF_019279795.1
lungfish	*Protopterus annectens*	KRT-T1-10	XP_043910901.1	GCF_019279795.1
lungfish	*Protopterus annectens*	KRT-T1-12	XP_043910895.1	GCF_019279795.1
lungfish	*Protopterus annectens*	KRT-T1-13	XP_043910893.1	GCF_019279795.1
lungfish	*Protopterus annectens*	KRT23L1	XP_043910879.1	GCF_019279795.1
lungfish	*Protopterus annectens*	KRT23L2	XP_043910874.1	GCF_019279795.1
lungfish	*Protopterus annectens*	KRT70	XP_043910867.1	GCF_019279795.1
lungfish	*Protopterus annectens*	KRT18	XP_043939780.1	GCF_019279795.1
lungfish	*Protopterus annectens*	KRT18L	XP_043939781.1	GCF_019279795.1

* GenBank, Axolotl-omics and preliminary names for previously unnamed genes:
keratin (KRT) - type I (T1) - sequential number according to position in the locus.
** Accession numbers refer to NCBI GenBank for all species except for axolotl.
Sequences of axolotl are from Axolotl-omics (https://www.axolotl-omics.org/assemblies).

Supplementary Table 2. Type II keratins used for phylogenetic analysis

Species (simplified name)	Species (scientific name)	Gene *	Accession number**	Genome version
human	*Homo sapiens*	KRT80	NP_872313	GRCh38.p14
human	*Homo sapiens*	KRT7	XP_011536627	GRCh38.p14
human	*Homo sapiens*	KRT81	NP_002272	GRCh38.p14
human	*Homo sapiens*	KRT86	XP_005268923	GRCh38.p14
human	*Homo sapiens*	KRT83	NP_002273	GRCh38.p14
human	*Homo sapiens*	KRT85	NP_002274	GRCh38.p14
human	*Homo sapiens*	KRT84	NP_149034	GRCh38.p14
human	*Homo sapiens*	KRT82	NP_149022	GRCh38.p14
human	*Homo sapiens*	KRT75	NP_004684	GRCh38.p14
human	*Homo sapiens*	KRT6b	NP_005546	GRCh38.p14
human	*Homo sapiens*	KRT6c	NP_775109	GRCh38.p14
human	*Homo sapiens*	KRT6a	NP_005545	GRCh38.p14
human	*Homo sapiens*	KRT5	NP_000415	GRCh38.p14
human	*Homo sapiens*	KRT71	XP_047284152	GRCh38.p14
human	*Homo sapiens*	KRT74	NP_778223.2	GRCh38.p14
human	*Homo sapiens*	KRT72	XP_047284279	GRCh38.p14
human	*Homo sapiens*	KRT73	XP_047284717	GRCh38.p14
human	*Homo sapiens*	KRT2	NP_000414	GRCh38.p14
human	*Homo sapiens*	KRT1	NP_006112	GRCh38.p14
human	*Homo sapiens*	KRT77	NP_778253.2	GRCh38.p14
human	*Homo sapiens*	KRT76	NP_056932	GRCh38.p14
human	*Homo sapiens*	KRT3	NP_476429	GRCh38.p14
human	*Homo sapiens*	KRT4	NP_002263	GRCh38.p14
human	*Homo sapiens*	KRT79	NP_787028	GRCh38.p14
human	*Homo sapiens*	KRT78	XP_011536312	GRCh38.p14
human	*Homo sapiens*	KRT8	NP_001243211	GRCh38.p14
human	*Homo sapiens*	KRT18	NP_954657	GRCh38.p14
frog	*Xenopus tropicalis*	krt80	NP_001163982	UCB_Xtro_10.0
frog	*Xenopus tropicalis*	krt7	XP_002935788	UCB_Xtro_10.0
frog	*Xenopus tropicalis*	krt59	XP_012813652	UCB_Xtro_10.0
frog	*Xenopus tropicalis*	krt6b	XP_031753073.1	UCB_Xtro_10.0
frog	*Xenopus tropicalis*	krt61	NP_001072377	UCB_Xtro_10.0
frog	*Xenopus tropicalis*	krt62	XP_002935776	UCB_Xtro_10.0
frog	*Xenopus tropicalis*	krt78.1	XP_002935786	UCB_Xtro_10.0
frog	*Xenopus tropicalis*	krt78.2	XP_002935775	UCB_Xtro_10.0
frog	*Xenopus tropicalis*	krt78.3	XP_002935785	UCB_Xtro_10.0
frog	*Xenopus tropicalis*	krt78.4	XP_002935784	UCB_Xtro_10.0
frog	*Xenopus tropicalis*	krt78.5	XP_031753072	UCB_Xtro_10.0
frog	*Xenopus tropicalis*	krt78.10	XP_031752089	UCB_Xtro_10.0
frog	*Xenopus tropicalis*	krt78.6	XP_002935774	UCB_Xtro_10.0
frog	*Xenopus tropicalis*	krt78.7	NP_001006716	UCB_Xtro_10.0
frog	*Xenopus tropicalis*	krt78.8	XP_031753071	UCB_Xtro_10.0
frog	*Xenopus tropicalis*	krt78.9	NP_001166034	UCB_Xtro_10.0

frog	*Xenopus tropicalis*	krt8	NP_001002797	UCB_Xtro_10.0
frog	*Xenopus tropicalis*	krt18	NP_988944	UCB_Xtro_10.0
axolotl	*Ambystoma mexicanum*	krt80	AMEX60DD201029833.1	AmexG_v6.0-DD
axolotl	*Ambystoma mexicanum*	krt7	AMEX60DD201029832.1	AmexG_v6.0-DD
axolotl	*Ambystoma mexicanum*	krt59	AMEX60DD301029825.1	AmexG_v6.0-DD
axolotl	*Ambystoma mexicanum*	krt61.1	AMEX60DD201029824.1	AmexG_v6.0-DD
axolotl	*Ambystoma mexicanum*	krt61.2	AMEX60DD201029823.1	AmexG_v6.0-DD
axolotl	*Ambystoma mexicanum*	krt78L1	AMEX60DD301029822.6	AmexG_v6.0-DD
axolotl	*Ambystoma mexicanum*	krt78L2	AMEX60DD201029819.9	AmexG_v6.0-DD
axolotl	*Ambystoma mexicanum*	krt78L3	AMEX60DD201029817.1	AmexG_v6.0-DD
axolotl	*Ambystoma mexicanum*	krt78L4	AMEX60DD301029816.2	AmexG_v6.0-DD
axolotl	*Ambystoma mexicanum*	krt78L6	AMEX60DD301029813.1	AmexG_v6.0-DD
axolotl	*Ambystoma mexicanum*	krt78L7	AMEX60DD201029812.1	AmexG_v6.0-DD
axolotl	*Ambystoma mexicanum*	krt8	AMEX60DD102029811.2	AmexG_v6.0-DD
axolotl	*Ambystoma mexicanum*	krt18	AMEX60DD201029807.1	AmexG_v6.0-DD
caecilian	*Rhinatrema bivittatum*	krt80.1	XP_029450891	aRhiBiv1.1
caecilian	*Rhinatrema bivittatum*	krt7	XP_029450895	aRhiBiv1.1
caecilian	*Rhinatrema bivittatum*	krt61	XP_029450896	aRhiBiv1.1
caecilian	*Rhinatrema bivittatum*	krt78L1	XP_029452691	aRhiBiv1.1
caecilian	*Rhinatrema bivittatum*	krt78L2	XP_029450898	aRhiBiv1.1
caecilian	*Rhinatrema bivittatum*	krt78L3	XP_029450897	aRhiBiv1.1
caecilian	*Rhinatrema bivittatum*	krt78L4	XP_029452692	aRhiBiv1.1
caecilian	*Rhinatrema bivittatum*	krt78L5	XP_029450901	aRhiBiv1.1
caecilian	*Rhinatrema bivittatum*	krt78L6	XP_029450902	aRhiBiv1.1
caecilian	*Rhinatrema bivittatum*	krt78L7	XP_029450903	aRhiBiv1.1
caecilian	*Rhinatrema bivittatum*	krt8	XP_029450904	aRhiBiv1.1
caecilian	*Rhinatrema bivittatum*	krt18	XP_029450905	aRhiBiv1.1
lungfish	*Protopterus annectens*	KRT80L1	XP_043939767.1	GCF_019279795.1
lungfish	*Protopterus annectens*	KRT80L2	XP_043939768.1	GCF_019279795.1
lungfish	*Protopterus annectens*	KRT-T2-1	XP_043939769.1	GCF_019279795.1
lungfish	*Protopterus annectens*	KRT-T2-2	XP_043939771.1	GCF_019279795.1
lungfish	*Protopterus annectens*	KRT-T2-3	XP_043939773.1	GCF_019279795.1
lungfish	*Protopterus annectens*	KRT-T2-4	XP_043939774.1	GCF_019279795.1
lungfish	*Protopterus annectens*	KRT-T2-5	XP_043939775.1	GCF_019279795.1
lungfish	*Protopterus annectens*	KRT-T2-6	XP_043939777.1	GCF_019279795.1
lungfish	*Protopterus annectens*	KRT-T2-7	XP_043939778.1	GCF_019279795.1
lungfish	*Protopterus annectens*	KRT-T2-8	XP_043939779.1	GCF_019279795.1

* GenBank, Axolotl-omics and preliminary names for previously unnamed genes:
keratin (KRT) - type II (T2) - sequential number according to position in the locus
** Accession numbers refer to NCBI GenBank for all species except for axolotl.
Sequences of axolotl are from Axolotl-omics (https://www.axolotl-omics.org/assemblies).
