# Supplementary material for: Evolutionary origin of Hoxc13-dependent skin appendages in amphibians
Source: Nat Commun. 2024 Mar 18;15:2328. doi: 10.1038/s41467-024-46373-x (PMC10948813; doi:10.1038/s41467-024-46373-x)
Supplement: Supplementary file 2 — Reporting Summary [file 41467_2024_46373_MOESM2_ESM.pdf]

nature portfolio

Corresponding author(s): Kris Vleminckx, Leopold Eckhart

Last updated by author(s): Feb 21, 2024

Reporting Summary

Nature Portfolio wishes to improve the reproducibility of the work that we publish. This form provides structure for consistency and transparency in reporting. For further information on Nature Portfolio policies, see our Editorial Policies and the Editorial Policy Checklist.

Statistics

For all statistical analyses, confirm that the following items are present in the figure legend, table legend, main text, or Methods section.

n/a	Confirmed	
☐	☒	The exact sample size (*n*) for each experimental group/condition, given as a discrete number and unit of measurement
☐	☒	A statement on whether measurements were taken from distinct samples or whether the same sample was measured repeatedly
☐	☒	The statistical test(s) used AND whether they are one- or two-sided<br>*Only common tests should be described solely by name; describe more complex techniques in the Methods section.*
☒	☐	A description of all covariates tested
☐	☒	A description of any assumptions or corrections, such as tests of normality and adjustment for multiple comparisons
☐	☒	A full description of the statistical parameters including central tendency (e.g. means) or other basic estimates (e.g. regression coefficient) AND variation (e.g. standard deviation) or associated estimates of uncertainty (e.g. confidence intervals)
☐	☒	For null hypothesis testing, the test statistic (e.g. *F*, *t*, *r*) with confidence intervals, effect sizes, degrees of freedom and *P* value noted<br>*Give P values as exact values whenever suitable.*
☒	☐	For Bayesian analysis, information on the choice of priors and Markov chain Monte Carlo settings
☐	☒	For hierarchical and complex designs, identification of the appropriate level for tests and full reporting of outcomes
☒	☐	Estimates of effect sizes (e.g. Cohen's *d*, Pearson's *r*), indicating how they were calculated

Our web collection on statistics for biologists contains articles on many of the points above.

Software and code

Policy information about availability of computer code

Data collection	Images were taken with an Olympus BX63 light microscope equipped with a UC-90 camera and the software cellSense Dimension (version: 2.3.18987.0, https://www.olympus-lifescience.com/en/software/cellsens/). qRT-PCR was performed on a Light Cycler® 480 II (Roche) equipped with the software LightCycler480 Software (version: 1.5, https://lifescience.roche.com/global/en/products/product-category/lightcycler.html).
Data analysis	For data analysis, we used CRISPRScan (https://www.crisprscan.org/), Indelphi (https://indelphi.giffordlab.mit.edu/), BATCH-GE (https://github.com/WouterSteyaert/BATCH-GE), CRISPResso2 (version: 2.2.1)(https://github.com/pinellolab/CRISPResso2), Proteome Discoverer Software 2.4.1.15 (https://www.thermofisher.com/at/en/home/industrial/mass-spectrometry/liquid-chromatography-mass-spectrometry-lc-ms/lc-ms-software/multi-omics-data-analysis/proteome-discoverer-software.html?gclid=Cj0KCQjw1_SkBhDwARIsANbGpFv76TBs8B3djD9BLIgLHMOOhFgATOWAxnJD4dSG2yvZIaGKXPh1zDwaAu1iEALw_wcB&cid=E.23CMD.DL103.12911.01&ef_id=Cj0KCQjw1_SkBhDwARIsANbGpFv76TBs8B3djD9BLIgLHMOOhFgATOWAxnJD4dSG2yvZIaGKXPh1zDwaAu1iEALw_wcB:G:s&s_kwcid=AL!3652!3!334040549172!p!!g!!proteome%20discoverer&gad=1), JASPAR (https://jaspar.genereg.net/), Prottest (version 3.0)(https://github.com/ddarriba/prottest3), PhyML (Version 20120412)(https://github.com/stephaneguindon/phyml), FigTree (version: 1.4.3)(http://tree.bio.ed.ac.uk/software/figtree/), Inkscape (version: 1.0.0.0)(https://inkscape.org/de/), GraphPad Prism 8 (version 8.0.1)(https://www.graphpad.com/features), Salmon (version: 1.10.1)(https://combine-lab.github.io/salmon/getting_started/), Python ((version: 3.10))(https://www.python.org/), and PyDESeq2 (version: 0.4.4)(https://pydeseq2.readthedocs.io/en/latest/).

For manuscripts utilizing custom algorithms or software that are central to the research but not yet described in published literature, software must be made available to editors and reviewers. We strongly encourage code deposition in a community repository (e.g. GitHub). See the Nature Portfolio guidelines for submitting code & software for further information.

Data

Policy information about availability of data

All manuscripts must include a data availability statement. This statement should provide the following information, where applicable:

- Accession codes, unique identifiers, or web links for publicly available datasets
- A description of any restrictions on data availability
- For clinical datasets or third party data, please ensure that the statement adheres to our policy

The mass spectrometry-based proteomic data generated in this study have been deposited into the ProteomeXchange Consortium via the PRIDE [53] partner repository (https://www.ebi.ac.uk/pride/) under accession code PXD041765 [https://www.ebi.ac.uk/pride/archive/projects/PXD041765]. The RNA-seq data generated in this study have been deposited on NCBI under accession code PRJNA1055414 [https://www.ncbi.nlm.nih.gov/bioproject/PRJNA1055414]. Accession codes/sources for publicly available original sequence data used in this study are provided in the Supplementary Information. All data needed to evaluate the conclusions in the paper are present in the paper and/or the Supplementary Materials. Source data are provided with this paper. The multiple sequence alignments for phylogeny generated in this study are provided in the Source Data file. The X. tropicalis reference genome UCB_Xtro_10.0 used as Reference database for the RNA-seq analysis is available on NCBI under accession number: GCA_000004195.4 [https://www.ncbi.nlm.nih.gov/datasets/genome/GCF_000004195.4/]. Source data are provided with this paper.

Research involving human participants, their data, or biological material

Policy information about studies with human participants or human data. See also policy information about sex, gender (identity/presentation), and sexual orientation and race, ethnicity and racism.

Reporting on sex and gender	The research did not include human participants or human data.
Reporting on race, ethnicity, or other socially relevant groupings	The research did not include human participants or human data.
Population characteristics	The research did not include human participants or human data.
Recruitment	The research did not include human participants or human data.
Ethics oversight	The research did not include human participants or human data.

Note that full information on the approval of the study protocol must also be provided in the manuscript.

Field-specific reporting

Please select the one below that is the best fit for your research. If you are not sure, read the appropriate sections before making your selection.

☒ Life sciences ☐ Behavioural & social sciences ☐ Ecological, evolutionary & environmental sciences

For a reference copy of the document with all sections, see nature.com/documents/nr-reporting-summary-flat.pdf

Life sciences study design

All studies must disclose on these points even when the disclosure is negative.

Sample size	Sample sizes were chosen based on the hypothesis that the effect of loss of Hoxc13 on claw formation in frogs would be as strong as the effect of loss of Hoxc13 on hair growth in mammals, so that sample sizes of 3-5 sufficed to test for significance of differences in morphology and gene expression.
Data exclusions	No data were excluded from analyses.
Replication	All experiments of quantification were performed in triplicates or quadruplicates. All attempts of replication were successful.
Randomization	Randomization was not implemented because allocation into experimental groups was not relevant to this study.
Blinding	Blinding was not required because the experiments, such as histological analysis, quantitative PCR, RNA-seq, and proteomics, were not sensitive to bias.

Reporting for specific materials, systems and methods

We require information from authors about some types of materials, experimental systems and methods used in many studies. Here, indicate whether each material, system or method listed is relevant to your study. If you are not sure if a list item applies to your research, read the appropriate section before selecting a response.

Materials & experimental systems

n/a	Involved in the study	
	x	Antibodies
	x	Eukaryotic cell lines
x		Palaeontology and archaeology
	x	Animals and other organisms
x		Clinical data
x		Dual use research of concern
x		Plants

Methods

n/a	Involved in the study	
x		ChIP-seq
x		Flow cytometry
x		MRI-based neuroimaging

Antibodies

Antibodies used	Anti-digoxigenin antibody (Fab fragments) conjugated to alkaline phosphatase (1:5000 dilution, catalog number: 11093274910, lot number 54732420, Roche)
Validation	Anti-digoxigenin AP-conjugate was validated in published study: Komatsu Y et al. Methods Mol Biol. 2014;1092:1-15. doi: 10.1007/978-1-60327-292-6_1.

Eukaryotic cell lines

Policy information about cell lines and Sex and Gender in Research

Cell line source(s)	HEK293T cells (female) were obtained from Mark Hall, Department of Biochemistry, University of Birmingham, Edgboston, Birmingham, B15 2 TT, UK (31/07/1996)
Authentication	STR profiling of the cells was described in Lin et al., Nat Commun. 2014;5:4767. DOI: 10.1038/ncomms5767
Mycoplasma contamination	The cell line tested negative for mycoplasma contamination.
Commonly misidentified lines (See ICLAC register)	No commonly misidentified cell lines were used in this study.

Animals and other research organisms

Policy information about studies involving animals; ARRIVE guidelines recommended for reporting animal research, and Sex and Gender in Research

Laboratory animals	This study involved Xenopus tropicalis frogs which were reared and bred in the animal facility of the University of Gent. Both male and female frogs were investigated. The aged of the investigated frogs ranges from 4-7 months.
Wild animals	No wild animals were included.
Reporting on sex	Both male and female frogs were investigated. No differences with regard to claw formation or gene expression were observed.
Field-collected samples	No field-collected samples were used.
Ethics oversight	Approval of the project was obtained by the Ethical Committee for Animal Experimentation, Ghent University, Faculty of Sciences (approval number EC2023-041).<br>Wildtype Xenopus tropicalis frogs from commercial providers were killed and tissues were sampled in accordance with the guidelines of the Ethics Committee of the Medical University of Vienna.

Note that full information on the approval of the study protocol must also be provided in the manuscript.
